# The relationship between weight and pulmonary outcomes in overweight and obese people with cystic fibrosis: A retrospective observational study

**DOI:** 10.1002/hsr2.910

**Published:** 2022-10-28

**Authors:** John J. Welter, Alison T. Lennox, Sankaran Krishnan, Christy Kim, Sheila Krishnan, Haley Thompson, Emily McAllister, Kristen Huang, Kasiemobi Nwaedozie, Allen J. Dozor

**Affiliations:** ^1^ Division of Pediatric Pulmonology New York Medical College Valhalla New York USA; ^2^ Touro College of Osteopathic Medicine New York New York USA

**Keywords:** body mass index, forced expiratory volume in 1 s, forced vital capacity, pulmonary exacerbations

## Abstract

**Background:**

A major focus in cystic fibrosis (CF) care aims to increase weight gain. Rates of overweight and obese people with CF have gradually increased over the past decade. Obesity could be a risk for restriction of lung volumes and airway obstruction as well as increase rates of pulmonary exacerbations in people with CF.

**Aim:**

To assess the relationship between weight categories and pulmonary outcomes in children and adults with CF.

**Methods:**

Patients 6 years of age and older were categorized into weight categories based on the Centers for Disease Control and Prevention (CDC) definitions. A retrospective chart review was conducted to obtain lung function testing and other outcomes.

**Results:**

One hundred five patients with a median age of 20.6 years were included in this analysis. 8.4%, 64%, 18%, and 10% of patients were underweight, normal/healthy weight, overweight, and obese, respectively. Forced expiratory volume in 1 s (FEV_1_) and forced vital capacity (FVC) (% predicted) did not differ between patients with weights in the normal range versus patients in the overweight/obese categories. Linear regression analysis showed a direct correlation between body mass index (BMI) and FEV_1_ that continued as BMI entered overweight and obese categories in both pediatric and adult patients. Overweight/obese patients did not have increased rates of pulmonary exacerbations compared to those in the normal/healthy weight category.

**Conclusion:**

As CF therapies continue to improve, an increasing number of people with CF are exceeding the CDC's normal‐weight range. Gaining weight past the normal range does not appear to negatively impact pulmonary health of people with CF. If this trend of increased weight gain continues, it remains to be seen if it will eventually negatively affect lung health.

## 1 INTRODUCTION

Cystic fibrosis (CF) is a disease characterized by chronic lung inflammation and malabsorption which may lead to failure to thrive in infancy without treatment.^1^ CF was first described in 1938 as CF of the pancreas,^2^ and before the advent of pancreatic enzyme replacement therapy, infants with CF would often die from malnutrition.^3^ Once pancreatic enzyme replacement became available, patients continued to have difficulties gaining weight due to increased metabolic demand from chronic inflammatory lung disease as well as inefficiencies of pancreatic enzyme replacement.^4^ Over the years, it has been demonstrated that nutritional status in people with CF, defined as percent predicted weight‐for‐length or body mass index (BMI), directly correlates with pulmonary function.^5^ This has made increased weight gain a major focus within the CF community. The CF Foundation has set a goal for pediatric patients of a BMI in the 50th percentile or greater, with patients defined as being at risk for nutritional failure with a BMI between 10th and 25th percentile, and in nutritional failure with a BMI below the 10th percentile.^6^ Recognition of the relationship between BMI and pulmonary function has led to increased efforts to promote weight gain.^7^ In 2009, for the first time in the United States, more than half of all people with CF between 2 and 19 years of age had a BMI greater than the 50th percentile.^7^ By 2017, this increased to almost 60%.^7^ The median BMI percentile has continued to rise in this population, and with it the proportion in overweight and obese categories has also continued to rise.^8^

Both otherwise healthy children and children with asthma who are obese have a higher forced vital capacity (FVC) compared to normal‐weight peers, with some evidence of airflow obstruction, such as reduced forced expiratory volume in 1 s (FEV_1_)/FVC and increased respiratory system resistance estimated with impulse oscillometry.^9^, ^10^, ^11^ In patients with asthma, obesity is associated with disease severity, and in adults, weight reduction has been shown to improve asthma severity and control, pulmonary function, and quality of life.^10^, ^12^

The effects of obesity on pulmonary outcomes in people with CF have not been adequately studied. In a study of overweight and obese adults with CF from 2015 to 2017, FEV_1_% predicted was directly related to rising BMI as patients entered the overweight, but not obese, categories.^13^ The objectives of this study were to assess the relationship between weight categories and pulmonary outcomes including pulmonary function and rates of pulmonary exacerbations in children and adults with CF.

## 2 METHODS

This is a single‐center retrospective observational study. A chart review was performed of patients with CF seen at the Armond V. Mascia, MD, Cystic Fibrosis Center at New York Medical College from July 1, 2014, to June 30, 2019. Patients were included if they were 6 years of age and older, had a sweat chloride greater than 60 mmol/L or 2 known CF‐causing mutations, and were able to meet american thoracic society criteria for adequate spirometry.^14^ Patients were excluded if who had undergone any solid organ transplant or were pregnant/lactating. BMI was calculated as weight (kg)/height (m^2^). Adults (people over 20 years of age) and children (people 2–20 years of age) were classified based on Centers for Disease Control and Prevention (CDC) criteria for weight distribution based on BMI percentiles or absolute BMI:

- Underweight: Children with BMI below 5th percentile, adults with BMI below 18.5.
- Normal or healthy weight: Children with BMI 5th to less than the 85th percentile, adults with BMI 18.5–24.9.
- Overweight: Children with BMI 85th to less than the 95th percentile, adults with BMI 25.0–29.9.
- Obese: Children with BMI equal to or greater than 95th percentile, adults with BMI 30.0 and above.

The presence of cystic fibrosis‐related diabetes (CFRD) was determined based on CF Foundation guidelines.^15^ Results from spirometry were expressed as % predicted values using global lung initiative norms.^16^ Individual FEV_1_% predicted, FVC% predicted, and the absolute ratio of FEV_1_/FVC were calculated as the median of the maximum value of each measure per quarter over the entire study. Pulmonary exacerbations were defined as the prescription of oral or intravenous antibiotics by the CF clinician for acute respiratory symptoms. Individual annual pulmonary exacerbation rates were calculated by dividing the number of pulmonary exacerbations over the course of the study by the number of years the patient participated in the study.

### 2.1 Statistical analysis

For the primary analysis, people in each of the CDC weight categories were compared. Patient characteristics including sex, sweat chloride levels, presence of CFRD, treatment with nutritional therapies, treatment with CFTR modulators, and pulmonary outcomes were evaluated for differences among weight categories. These characteristics and CDC weight categories were compared between children (6–19 years of age) and adults (greater than or equal to 20 years of age). Categorical values were summarized by frequencies and were compared using *χ* ^2^ analysis. Continuous variables were summarized by median and interquartile range (IQR) and were compared using the Mann–Whitney rank sum test. For the secondary analysis, linear regression was performed in children and adults, comparing BMI percentiles and absolute BMI with FEV_1_% predicted and FVC% predicted. Two‐sided *p* < 0.05 were considered statistically significant. All statistical analyses were performed using SigmaPlot software, version13.0 (Systat Software Inc.).

## 3 RESULTS

### 3.1 Patient characteristics

Patient characteristics and comparison of weight categories are in Table 1. One Hundred and five people with CF, 6 years of age or older, seen over the 5‐year study period, completed 1931 clinic visits that were included in this analysis. Median age was 20.5 years (IQR: 11.9, 25.7). 9%, 64%, 18%, and 10% of patients were underweight, normal/healthy weight, overweight, and obese, respectively. There were no statistically significant changes in the proportion of patients in each weight category over the 5 years evaluated. Obese patients had significantly lower sweat chloride (71 vs. 88, 97, and 91 mmol/L, *p* = 0.008) and were less likely to be prescribed pancreatic enzymes (30% vs. 89%, 87%, and 95%, *p* < 0.001) than people who were underweight, normal weight, and overweight, respectively. More obese patients were prescribed ivacaftor than those with normal/healthy weight (50% vs. 15%, respectively, *p* = 0.02). We did not find a difference in ivacaftor/tezacaftor or ivacaftor/lumacaftor use between groups. Underweight people were more likely to have CFRD than normal/healthy weight and obese people (56% vs. 10% and 0%, respectively, *p* = 0.004 and 0.01).

**Table 1:** Comparison of patient characteristics in different weight categories

	Overall	Underweight^a^	Normal/healthy weight^b^	Overweight^c^	Obese^d^	*p* Value between all four weight categories
*N*	105	9 (9%)	67 (64%)	19 (18%)	10 (10%)	
Age, years, (median, IQR)	20.5 (11.9, 25.7)	20.5 (14.4, 25.1)	18.9 (11.3, 24.0)	22.7 (13.4, 29.6)	20.6 (9.3, 26.6)	0.21
Female, *N* (%)	48 (46%)	6 (7%)	30 (45%)	7 (37%)	5 (50%)	0.51
Sweat chloride (mmol/L) (median, IQR)	94 (83, 104)	88.0 (81.5, 94.0)	97.0 (88.8, 104.6)	91.0 (82.5, 102.5)	71.0 (68.5, 87.5)	0.008
Pancreatic enzyme replacement, *N* (%)	87 (83%)	8 (89%)	58 (87%)	18 (95%)	3 (30%)	<0.001
Oral, nasogastric, or gastrostomy tube supplemental feedings, *N* (%)	39 (37%)	6 (67%)	29 (43%)	2 (11%)	2 (20%)	0.009
CFRD, *N* (%)	16 (15%)	5 (56%)	7 (10%)	4 (21%)	0 (0%)	0.002
Insulin use, *N* (%)	10 (10%)	3 (33%)	5 (7%)	2 (11%)	0 (0%)	0.062
Ivacaftor	19 (18%)	1 (11%)	10 (15%)	3 (16%)	5 (50%)	0.053
Ivacaftor/tezacaftor or ivacaftor/lumacaftor, *N* (%)	37 (35%)	5 (56%)	18 (27%)	10 (53%)	4 (40%)	0.098
Annual pulmonary exacerbation rate (median, IQR)	1.0 (0.63, 1.60)	1.0 (0.53, 1.88)	1.2 (0.60, 1.80)	1.0 (0.67, 1.33)	1.3 (0.63, 1.60)	0.93
FEV_1_, % predicted (median, IQR)	81.2 (62.3, 92.2)	49.0 (33.0, 68.5)	80.7 (65.0, 91.3)	83.7 (68.5, 93.0)	92.0 (83.6, 102.9)	0.002
FVC, % predicted (median, IQR)	88.0 (76.9, 101.0)	76.5 (48.1, 84.9)	88.0 (78.4, 100.4)	93.7 (76.3, 108.9)	93.3 (86.8, 105.3)	0.051
FEV_1_/FVC (median, IQR)	0.79 (0.70, 0.85)	0.62 (0.41, 0.78)	0.80 (0.70, 0.85)	0.77 (0.65, 0.84)	0.83 (0.79, 0.90)	0.008
FEF25–75, % predicted (median, IQR)	67.7 (42.5, 91.8)	31.6 (11.3, 47.8)	62.7 (36.7, 86.6)	71.0 (31.6, 91.9)	80.5 (66.0, 103.3)	0.004

Abbreviations: BMI, body mass index; CFRD, cystic fibrosis‐related diabetes; FEV_1_, forced expiratory volume in 1 s; FVC, forced vital capacity; IQR, interquartile range.

^a^ Children with BMI below 5th percentile, adults with BMI below 18.5.

^b^ Children with BMI 5th to less than the 85th percentile, adults with BMI 18.5–24.9.

^c^ Children with BMI 85th to less than the 95th percentile, adults with BMI 25.0–29.9.

^d^ Children with BMI equal to or greater than 95th percentile, adults with BMI 30.0 and above.

Children and adults are compared in Table 2. 19% of children and 35% of adults were characterized as either overweight or obese, *p* = 0.06. Children were more likely to be in the normal/healthy weight category than adults, *p* = 0.02. We did not find significant differences in gender, pancreatic enzyme replacement, sweat chloride, rates of CFRD or nutritional supplements between children and adults.

**Table 2:** Comparison between children and adults with CF

	Overall	Children (6–19 years of age)	Adults (≥20 years of age)	Children versus adults *p* Value
*N*	105	48 (46%)	57 (54%)	N/A
Age, years, (median, IQR)	20.5 (11.9, 25.7)	11.4 (8.5, 14.8)	24.8 (22.3, 29.5)	<0.001
Female, *N* (%)	48 (46%)	25 (52%)	23 (4%)	0.32
Pancreatic enzyme replacement, *N* (%)	87 (83%)	38 (79%)	49 (86%)	0.51
Sweat Chloride (median, IQR)	94 (83, 104)	92.8 (78.3, 86.0)	96.0 (86.0, 105.5)	0.51
CFRD, *N* (%)	16 (15%)	4 (8%)	12 (21%)	0.13
Insulin, *N* (%)	10 (10%)	2 (4%)	8 (14%)	0.17
Oral, nasogastric, or gastrostomy tube supplemental feedings, *N* (%)	39 (37%)	21 (44%)	18 (32%)	0.28
Underweight, *N* (%)^a^	9 (9%)	2 (4%)	7 (12%)	0.26
Normal/healthy weight, *N* (%)^b^	67 (64%)	37 (77%)	30 (53%)	0.02
Overweight, *N* (%)^c^	19 (18%)	2 (4%)	17 (30%)	0.002
Obese, *N* (%)^d^	10 (9%)	7 (15%)	3 (5%)	0.20
Annual exacerbation rate (median, IQR)	1.0 (0.63, 1.60)	1.0 (0.7, 1.6)	1.2 (0.6, 1.6)	0.76
FEV_1_% predicted (median, IQR)	81.2 (62.3, 92.2)	86.9 (77.6, 95.7)	70.8 (49.7, 87.8)	<0.001
FVC % predicted (median, IQR)	88.0 (76.9, 101.0)	93.0 (80.9, 103.8)	84.5 (73.2, 98.2)	0.052
FEV_1_/FVC (median, IQR)	0.79 (0.70, 0.85)	0.84 (0.80, 0.87)	0.72 (0.59, 0.80)	<0.001
FEF25–75% predicted (median, IQR)	67.7 (42.5, 91.8)	76.7 (57.9, 92.9)	45.9 (20.0, 74.4)	<0.001

Abbreviations: BMI, body mass index; CF, cystic fibrosis; CFRD, cystic fibrosis‐related diabetes; FEV_1_, forced expiratory volume in 1 s; FVC, forced vital capacity; IQR, interquartile range.

^a^ Children with BMI below 5th percentile, adults with BMI below 18.5.

^b^ Children with BMI 5th to less than the 85th percentile, adults with BMI 18.5–24.9.

^c^ Children with BMI 85th to less than the 95th percentile, adults with BMI 25.0–29.9.

^d^ Children with BMI equal to or greater than 95th percentile, adults with BMI 30.0 and above.

### 3.2 Pulmonary outcomes

**Figure 1:**
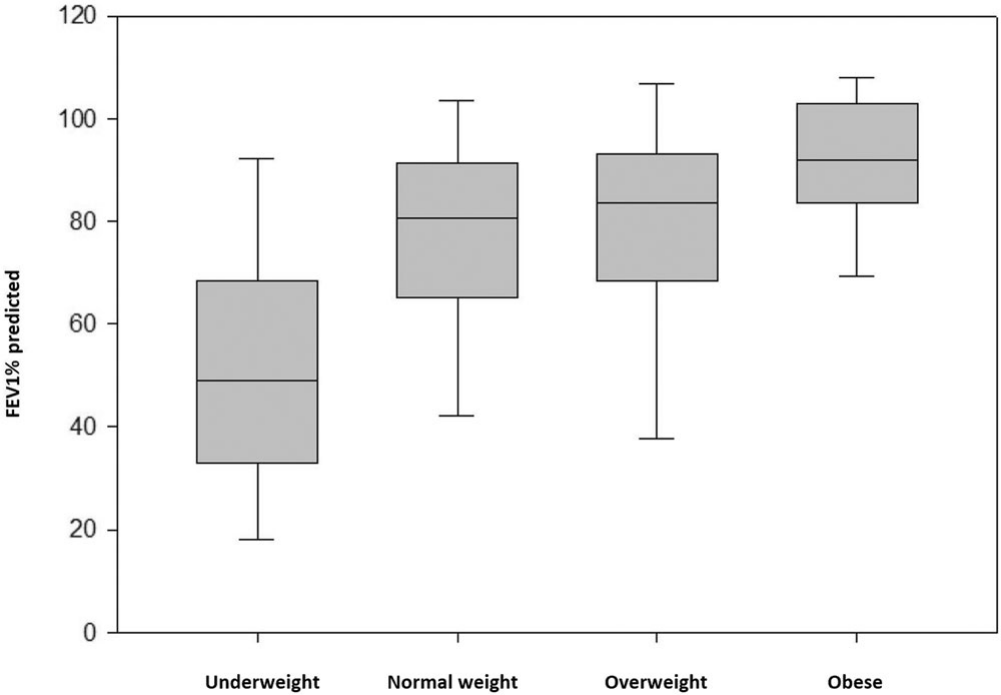
FEV_1_% predicted among CDC weight categories, all patients. Underweight = BMI < 18.5/less than 5th percentile, normal/health weight = BMI 18.5–24.9/5th to less than 85th percentile, overweight = BMI 18.5–24.9/5th to less than 85th percentile, obese = BMI 18.5–24.9/5th to less than 85th percentile. One‐way ANOVA on ranks, *p* = 0.002. The ends of the boxes define the 25th and 75th percentiles, with a line at the median and error bars defining the 10th and 90th percentiles. ANOVA, analysis of variance; BMI, body mass index; CDC, Centers for Disease Control and Prevention; FEV_1_, forced expiratory volume in 1 s.

**Figure 2:**
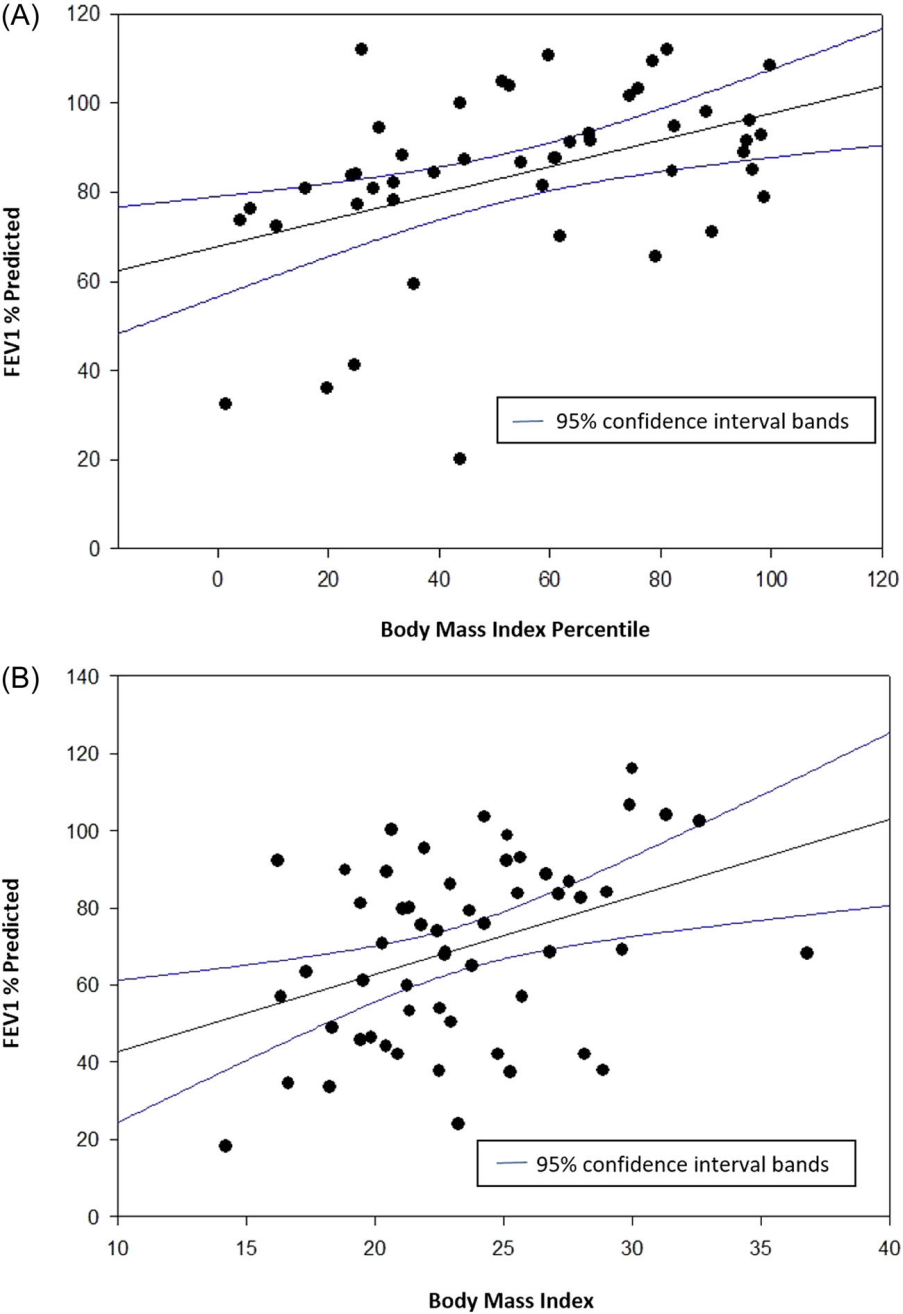
Body mass index versus FEV_1_% predicted in people with cystic fibrosis. (A) <20 years of age. *R* ^2^ = 0.19, *p* = 0.002. (B) >20 years of age. *R* ^2^ = 0.15, *p* = 0.003. FEV_1_, forced expiratory volume in 1 s. FEV_1_, forced expiratory volume in 1 s.

There were no statistically significant differences in annual rates of pulmonary exacerbations between weight categories (Table 1). FEV_1_% predicted FEV_1_/FVC and FEF25–75% predicted were lower in people who were underweight than those in the other weight categories (Table 1, Figure 1). Scattergrams of FEV_1_% predicted and BMI are presented in Figure 2. On linear regression, there was a direct correlation between BMI and FEV_1_% predicted in all weight categories in both children and adults (Figure 2A,B).

**Figure 3:**
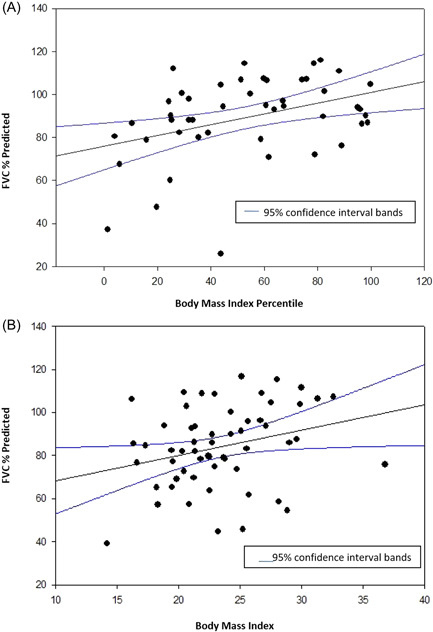
Body mass index versus FVC predicted in people with cystic fibrosis. (A) <20 years of age. *R* ^2^ = 0.153, *p* = 0.006. (B) >20 years of age. *R* ^2^ = 0.0781, *p* = 0.04. FVC, forced vital capacity.

The relationship between BMI and FVC is demonstrated in Figure 3. There was a direct correlation between BMI, percentile, and FVC, % predicted, in both children and adults.

## 4 DISCUSSION

It is well established that pulmonary function and nutritional status, as reflected by BMI, are highly correlated in people with CF.^17^ However, increasing rates of obesity in people with CF raise the concern that this relationship may not hold true at higher BMIs. There are known detrimental effects of obesity in other chronic lung diseases. Obese people with COPD have decreased lung compliance and reduced small airway flows and obese people with asthma have more frequent and severe exacerbations and are less responsive to treatment.^18^, ^19^ Asthma and COPD are similar to CF as they are all chronic obstructive lung diseases but differ in their underlying pathophysiology. Similar to Hanna et al.^20^ in a 2015 study, we found underweight people with CF have lower FEV_1_% predicted when compared to other weight groups (Table 1, Figure 1).^20^ In this single‐center study, the positive correlation between BMI and pulmonary function continued even in obese patients (see Figures 1, 2, 3). Furthermore, the rates of pulmonary exacerbations were not higher in obese patients (Table 1).

One might speculate that obesity could result in lung restriction, that is, decreased FVC, but this was not noted in this study. In people with CF, the greater the BMI, the greater the FVC. This is consistent with observations in healthy children and children with asthma, though the reasons for this finding are unclear.^11^ There was no evidence of restrictive lung disease in those who were obese. Vital capacity was reduced in underweight people with CF when compared to the other weight categories.

While we found people with CF who are obese have higher FEV_1_% predicted than underweight people with CF, we did not find a statistically significant increase in FEV_1_% predicted when obese people with CF were compared to those in the normal or overweight categories. There are multiple potential reasons why people with CF who are obese have higher FEV_1_% predicted than those who are underweight. Pancreatic enzyme use was significantly lower in the obese group. Pancreatic sufficiency and milder lung disease are associated with lower sweat chloride levels, as was seen in the obese group, likely due to relatively increased CFTR function and subsequently less severe disease.^21^ Underweight patients in this analysis were more likely to have CFRD. It is well known that CFRD is associated with decline in lung function as well as decreasing nutritional status in people with CF.^22^ While this study shows continued improvements in parameters associated with respiratory health in people with CF even as they become obese, there is insufficient evidence to support counseling patients to take steps to increase weight gain beyond the normal/healthy CDC weight category. It is not clear whether worsening rates, and severity, of obesity might eventually begin to have detrimental effects on pulmonary function in people with CF.

We found some evidence of higher rates of ivacaftor use in obese patients compared to the other weight categories, although these differences did not meet statistical significance. It is possible that the continued improvement of airway flows and volumes in obese patients could be attributed to the potentiation of lung CFTR activity by ivacaftor rather than simply the nutritional benefit. We did not find the same trend in those on ivacaftor/lumacaftor or ivacaftor/tezacaftor. By the end of our study ivacaftor/tezacaftor/elexacaftor has been approved for people with CF who have at least one F508del mutation, and it is possible that almost 90% of people with CF will have highly effective CFTR modulators (HEM) available for treatment. People with CF started on HEM have significant increases in weight and it is concerning that this may contribute to a rapid increase in obesity rates among people with CF.^23^, ^24^ Given potential increases in life expectancy with HEM use, these people may be at increased risk for obesity‐related diseases such as cardiovascular and endocrine disorders. Surprisingly, we found 4 (14%) of the 29 patients in the overweight and obese categories are still prescribed supplemental feedings, which highlights the continued focus on weight gain in patients with CF even after achieving healthy weight. Obesity increases the risk for comorbidities seen in the general population such as hypertension, obstructive sleep apnea, Type 2 diabetes, and cancer and can also lead to lower lung transplant survival rates in people with CF.^25^, ^26^, ^27^ Given the rising rates of obesity in people with CF, revision of nutritional guidelines may be required, especially in people prescribed HEM.

Our study has several limitations. This was a retrospective chart review and the historical data collected was limited by chart documentation which did not allow the collection of data such as caloric intake and diet quality. Our study includes both children and adults with CF who may have different rates of overweight and obesity, although we did not detect any difference in this study. CF center nutritional outcomes vary across the United States and between nations. Since this is a single‐center study, the proportion of patients categorized as either overweight or obese may not reflect the overall population of people with CF. Outcomes such as supplemental feedings were based on patient plans and may not reflect the actual number of patients adhering to these plans. Since this is a cross‐sectional study, causal relationships cannot be inferred between BMI and pulmonary outcomes. BMI was used to classify patients' nutritional status but body composition of fat and lean muscle mass may vary widely in people with the same BMI and may more directly impact pulmonary outcomes than BMI alone.

## 5 CONCLUSION

In this single‐center study, 28% of people with CF were overweight or obese. We did not find any adverse effects of obesity on pulmonary function or rate of pulmonary exacerbations over a 4‐year period. The introduction of highly effective CFTR modulators may be increasing the number of people with CF who are overweight and obese which could significantly alter nutritional guidance for these patients. If the current trend of increased weight gain in people with CF continues, it will remain to be seen if obesity may eventually negatively impact lung health.

## AUTHOR CONTRIBUTIONS

**John J. Welter**: Conceptualization; data curation; formal analysis; investigation; methodology; project administration; resources; supervision; writing – original draft; writing – review and editing. **Alison T. Lennox**: Conceptualization; methodology; project administration; resources; supervision; writing – review and editing. **Sankaran Krishnan**: Conceptualization; methodology; project administration; supervision; writing – review and editing. **Christy Kim**: Conceptualization; methodology; resources; supervision; writing – review and editing. **Sheila Krishnan**: Formal analysis; writing – review and editing. **Haley Thompson**: Investigation; writing – review and editing. **Emily McAllister**: Data curation; investigation; writing – review and editing. **Kristen Huang**: Data curation; investigation; writing – review and editing. **Kasiemobi Nwaedozie**: Data curation; investigation; writing – review and editing. **Allen J. Dozor**: Conceptualization; formal analysis; methodology; project administration; resources; supervision; writing – review and editing.

## CONFLICT OF INTEREST

The authors declare no conflict of interest.

## TRANSPARENCY STATEMENT

The lead author John J. Welter affirms that this manuscript is an honest, accurate, and transparent account of the study being reported; that no important aspects of the study have been omitted; and that any discrepancies from the study as planned (and, if relevant, registered) have been explained.

## ETHICS STATEMENT

The study was approved by the New York Medical College Institutional Review Board.

## Data Availability

The data that support the findings of this study are available from the corresponding author upon reasonable request. All authors have read and approved the final version of the manuscript, John Welter has full access to all of the data in this study and take complete responsibility for the integrity of the data and the accuracy of the data analysis.
